# New Peptide Based Fluconazole Conjugates with Expanded Molecular Targets

**DOI:** 10.3390/pharmaceutics14040693

**Published:** 2022-03-23

**Authors:** Wioletta Brankiewicz, Joanna Okońska, Katarzyna Serbakowska, Jan Lica, Marek Drab, Natalia Ptaszyńska, Anna Łęgowska, Krzysztof Rolka, Piotr Szweda

**Affiliations:** 1 Department of Pharmaceutical Technology and Biochemistry, Faculty of Chemistry, Gdańsk University of Technology, Narutowicza 11/12, 80-233 Gdańsk, Poland; wioletta.brankiewicz@pg.edu.pl (W.B.); s169840@student.pg.edu.pl (K.S.); 2 Department of Molecular Biochemistry, Faculty of Chemistry, University of Gdansk, 80-308 Gdańsk, Poland; joanna.okonska@phdstud.ug.edu.pl (J.O.); 24556@gumed.edu.pl (J.L.); anna.legowska@ug.edu.pl (A.Ł.); krzysztof.rolka@ug.edu.pl (K.R.); 3 Unit of Nanostructural Bio-Interactions, Hirszfeld Institute of Immunology and Experimental Therapy, Polish Academy of Sciences, 12 Weigla-Street, 53-114 Wrocław, Poland; marek.drab@hirszfeld.pl

**Keywords:** *Candida*, fluconazole, cell penetrating peptides, antimicrobial peptides, resistance, peptide-drug conjugates

## Abstract

Infections of *Candida* spp. etiology are frequently treated with azole drugs. Among azoles, the most widely used in the clinical scenario remains fluconazole (FLC). Promising results in treatment of dangerous, systemic *Candida* infections demonstrate the advantages of combined therapies carried out with combinations of at least two different antifungal agents. Here, we report five conjugates composed of covalently linked FLC and cell penetrating or antimicrobial peptide: TP10-7-NH_2_, TP10-NH_2_, LFcinB(2-11)-NH_2_, LFcinB[Nle^1,11^]-NH_2_, and HLopt2-NH_2_, with aspects of design, chemical synthesis and their biological activities. Two of these compounds, namely FLCpOH-TP10-NH_2_ and FLCpOH-TP10-7-NH_2_, exhibit high activity against reference strains and fluconazole-resistant clinical isolates of *C. albicans*, including strains overproducing drug transporters. Moreover, both of them demonstrate higher fungicidal effects compared to fluconazole. Analysis performed with fluorescence and scanning electron microscopy as well as flow cytometry indicated the cell membrane as a molecular target of synthesized conjugates. An important advantage of FLCpOH-TP10-NH_2_ and FLCpOH-TP10-7-NH_2_ is their low cytotoxicity. The IC_90_ value for the human cells after 72 h treatment was comparable to the MIC_50_ value after 24 h treatment for most strains of *C. albicans*. In reported conjugates, FLC was linked to the peptide by its hydroxyl group. It is worth noting that conjugation of FLC by the nitrogen atom of the triazole ring led to practically inactive compounds. Two compounds produced by us and reported herein appear to be potential candidates for novel antifungal agents.

## 1. Introduction

Effective and safe therapeutic treatment of fungal infections is a major challenge for modern medicine. This largely arises from the limitations of current antifungal agents with respect to host toxicity, effective drug delivery and the increasing incidence of antifungal resistance. Globally, it is estimated that nearly a billion humans have cutaneous fungal infections, many 10s of millions have mucosal candidiasis, and more than 150 million patients suffer from serious fungal diseases, among them candidiasis; infections caused by *Candida* spp. are the most common. The fungal infections have a major impact on quality of life and also exhibit high levels of mortality [1]. Candidiasis is typically of endogenous origin, occurring after debilitation of the individual following immunosuppressive and cytotoxic therapies, treatment with broad-spectrum antibiotics, or when accompanying other diseases such as AIDS or diabetes [2].

Compared to the large number and large diversity of chemical structures of antibacterial agents, the antifungal therapeutic options are still limited to only a few drug classes, namely polyenes, triazole derivatives, echinocandins, allylamines, and flucytosine [3], none of which fulfill all desired expectations. Polyenes are toxic and exhibit low water solubility, and echinocandins can only be administered intravenously, while allylamines such as terbinafine lack anticandidal activity. Currently, azoles are the most widely used class of antifungal drugs [4]. The mode of their activity is based on binding to and inhibiting lanosterol 14α-demethylase, a cytochrome P450 enzyme encoded by the ERG11 gene [5,6]. Inhibition of this enzyme leads to depletion of ergosterol from cell membranes. This affects the fluidity of the lipid bilayer and slows fungal growth. Moreover, inhibition of Erg11p also results in the accumulation of toxic metabolites such as 14-methyl-3,6-diol [7]. The combined effects of ergosterol depletion and toxic metabolite production are fungistatic for many pathogenic fungi.

Although many azoles have been synthesized, only some of them meet the criteria of an efficient and safe medicine that can be used in clinical practice. The first derivatives of imidazole, namely clotrimazole and miconazole, were introduced in the late 1960s. Due to unacceptable side effects following oral administration, at present clotrimazole usage is limited only to topical fungal infections; toxicity associated with intravenous application of miconazole caused its withdrawal from the market. In 1981, the Food and Drug Administration (FDA) approved ketoconazole, an imidazole derivative synthesized and developed by Janssen Pharmaceutica. For almost a decade, it was the only oral agent available for the treatment of systemic fungal infections. Unfortunately, therapy with this agent also resulted in several serious side effects including hepatotoxicity, a variety of endocrine disturbances (inhibition of testosterone and cortisol synthesis) and undesirable, often unpredictable, interactions with other drugs (e.g., cyclosporine) [4,8,9].

In the early 1990s, Pfizer developed fluconazole (FLC)—a triazole derivative that quickly became the antifungal agent of primary importance. Fluconazole is water-soluble, safe for patients even at a high daily dose (1600 mg–1.6 g), can be applied either intravenously or orally, and has a beneficial pharmacokinetic profile and broad spectrum of activity [9]. It effectively inhibits the growth of: *Candida albicans* and exhibits satisfactory activity against *C. tropicalis*, *C. parapsilosis*, *Candida lusitaniae*, *Candida dublinensis*, *Candida glabrata*, and *Candida guilerimondi,* while *Candida krusei* and particularly *Candida auris* exhibit higher levels of resistance [10,11,12]. The list of side effects of fluconazole usage is quite short; in fact, only some hepatotoxic effects are observed as a result of long-term therapies with this agent. The incidence rate of azole-induced liver injury remains low, about 2–4% for itraconazole, 2–10% for ketoconazole, and only 1% for fluconazole [13,14]. Due to these advantages, fluconazole is widely used not only for the treatment of candidiasis but also in prophylaxis, e.g., preventing invasive candidiasis in neonates [15] or *Candida vaginitis* [16]. However, fluconazole usage has developed some negative consequences, among which the most important are: (i) selection of fungal pathogens that exhibit lower susceptibility to fluconazole activity, including some non-albicans *Candida* spp. (e.g., *C. krusei*, *C. glabrata*); (ii) development of resistance against this agent among species that are naturally sensitive, e.g., *C. albicans*. The main mechanisms of azole resistance include alterations in the ERG11 gene (mutations and overexpression), which encodes the azole target enzyme, and upregulation of the genes coding for both ABC and MFS drug efflux transporters.

Efforts to overcome limitations of fluconazole resulted in new triazole derivatives; among them, voriconazole, posaconazole, itraconazole and isavuconazole seem to be the most promising. Some new derivatives, e.g., genaconazole, iodiconazole, saperconazole and oteseconazole, are at different stages of clinical trials; however, the availability of these substances is very limited [9]. Unfortunately, resistance often develops following usage of these new agents. Different approaches are proposed for improving antifungal efficiency and preventing development of resistance against this class of chemotherapeutics. Combined therapies carried out with combinations of at least two different antifungal agents (including different azoles), brought promising results in the treatment of dangerous, systemic *Candida* infections [17]. Moreover, following in vitro studies, several research groups reported positive effects of using azoles in combination with natural products or their constituents: resveratrol [18], eucalyptal D [19], magnolol [20] and propolis [21], which usually affect molecular targets other than enzymes involved in ergosterol synthesis within fungal cells. These combined therapies reduced the development of resistant mutants overproducing drug efflux pumps and/or modified versions of lanosterol 14α-demethylase. Another approach, that is proposed in this study, is modification of fluconazole’s (and other azoles’) structure by covalently linking it to other substances with different structures and biological activities.

**Figure 1:**
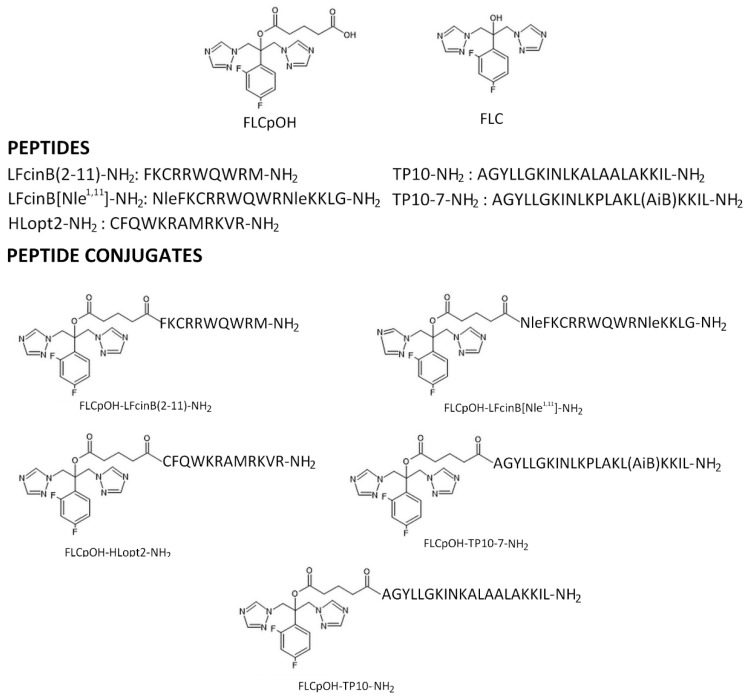
Chemical structures of synthesized conjugates and their constituents.

Herein we report the chemical synthesis and biological activity of conjugates of fluconazole with (i) cell-penetrating peptides (CPP), namely TP10-NH_2_ and TP10-7-NH_2_, or (ii) antimicrobial peptides (AMP), such as LFcinB(2-11)-NH_2_, LFcinB[Nle^1,11^]-NH_2_ and HLopt2-NH_2_. Both constituents of produced conjugates (FLC and peptides) display different modes of antifungal activity and affect different molecular targets within fungal cells. CPPs serving as carrier peptides and are considered the fundamental part of an extensively developed concept of a drug delivery system; TP-10 is one of the most promising peptides in this family. These peptides effectively penetrate cell membrane. In our recent paper [22], we showed that TP10-NH_2_ can be successfully applied to design conjugates with antimicrobial activity. Modified fragments of bovine lactoferrin (LFcin) and human lactoferrin (HLopt2) are members of the AMP family, peptides that constitute an important segment of the body’s natural immunity against microorganisms. We already proved that analogues of both lactoferrins are beneficial in designing antimicrobial conjugates [23,24,25]. The major foreseen advantages of conjugates of FLC and selected CPPs or AMPs include improved antifungal (hopefully fungicidal) efficiency and increased drug delivery. Moreover, we suspect that differences in cell uptake and structures of the conjugates compared to FLC alone could limit their efflux by ABC/MSF drug transporters. The outcomes of the study substantially confirmed our assumptions. Some of the produced conjugates show promising activity and are good candidates for obtaining new effective antifungals. Five conjugates, named here as FLCpOH-LFcinB(2-11)-NH_2_, FLCpOH-LFcinB[Nle^1,11^]-NH_2_, FLCpOH-HLopt2-NH_2_, FLCpOH-TP10-7-NH_2_ and FLCpOH-TP10-NH_2_, were synthesized. In all studied conjugates, glutaric acid served as a linker, forming covalent bonds between hydroxyl group of FLC and *N*-terminal amino group of the peptide (ester and amide moieties, respectively). Primary structures of the synthesized conjugates, together with constituent FLC derivative and the constituent peptides, are given in Figure 1.

## 2. Results and Discussion

### 2.1. Synthesis

Five peptide conjugates containing fluconazole were obtained (Figure 1). Their HPLC and mass spectrometry (MALDI-TOF) data as well as the total yields of the syntheses are shown in Table 1 and in Figure S1 (Supplementary Material). The physico-chemical characteristics of the fluconazole derivative (FLCpOH) used for coupling to the peptide and conjugate constituents is given in Table 1 and in Figure S1 of Supplementary Material. FLCpOH was obtained by the reaction of FLC with glutaric anhydride, applying microwave radiation. All peptides were synthesized by the solid phase method using Fmoc chemistry as described in our previous publications [23,25]. Fluconazole derivative was attached to the *N-*terminal amino group of peptidyl resin at the last step of the synthesis. Finally, conjugates were removed from the solid support together with deprotection of all functional groups in a one-step procedure, analyzed and purified by HPLC on reversed phase C18 analytical and semipreparative columns, respectively. In order to confirm the correctness of their structures, MS analyses of all conjugates and intermediate products were carried out. The selection of peptides for designing of FLC conjugates was based on the outcomes of our research to date and on the properties of these peptides, among which cell penetrating potential and antimicrobial activity were the most important. The TP10-NH_2_ has an amide moiety at the *C*-terminus and is produced by deletion of the *N-*terminal hexapeptide from transportan (TP). Transportan is a chimeric oligopeptide composed of the first 12 amino acid residues of the neuropeptide galanin and the 14-amino-acid-residue-long wasp venom peptide, mastoparan, connected via a lysine residue [26]. The shortened version of the peptide, TP10-NH_2_, retains high cell penetration potential and antimicrobial activity and additionally shows fewer side effects compared to TP. Its analogue, named as TP10-7-NH_2_, was reported to display improved properties with respect to antimicrobial activity and cytotoxicity [27]. Three other peptides used in this study were derived from the whey iron-binding glycoprotein lactoferrin (Lf). Enzymatic digestion of Lf releases several peptides that exhibit antimicrobial activity. Among them, LFcin is of special interest. HLopt2 is an analogue of the fragment 20–31 of human lactoferrin extended at *N*-terminus by Cys with Gln24 and Asn26 substituted for Lys and Ala, respectively. This fragment of human lactoferrin exerts antibacterial and bactericidal activity, which is mainly a consequence of the presence of an LPS-binding site. HLopt2 cause quick and complete loss of membrane potential. Conjugation of the antimicrobial drugs ciprofloxacin (CIP) and levofloxacin (LVX) with HLopt2 retained the antimicrobial activity of antibiotics while highly improving their solubility and helped to internalized them into both *C. albicans* and *S. aureus* cells [25]. In our previous work [23], we showed that modified bovine lactoferricin-truncated analogues (LFcinB), when conjugated with antimicrobial CIP or LVX, exerted, in contrast to these fluoroquinolones alone, activity against *Candida* yeast and were able to penetrate *C. albicans* cells. This interesting result motivated us to investigate conjugates with antifungal agents such as FLC. As already mentioned, both conjugates’ constituents (drug and peptide) are covalently linked by glutaric acid. While an amide bond on the peptide side is relatively stable in biological systems (far more so than peptide bonds that are specifically cleaved by ubiquitous proteinases), the ester moiety present on the FLC side is much more sensitive and can be cleaved by enzymes with esterase activity. Such an approach affords a new and very attractive possibility to design esterase-sensitive pro-drugs with FLC as an active part of the conjugate.

### 2.2. Anti-Candidal In Vitro Activity

The antifungal activity of the newly prepared fluconazole-based conjugates and the intermediate compound (FLCpOH) was first evaluated against a panel of twelve *C. albicans* (two reference strains and ten clinical isolates), *C. glabrata* (reference strain), and *C. krusei* (reference strain) strains using a concentration range of 8–250 µM (Table 2). Commercially available antifungal agents such as AmB (amphotericin B) and FLC were used as positive controls for comparison. For synthesized compounds and the reference drugs AmB and FLC, we reported MIC_90_ values, which corresponded to no visible growth of the fungal strains tested, and MIC_50,_ which corresponded to at least 50% growth inhibition compared with the control well. In order to determine fungicidal potential, we determined the value of the minimum fungicidal concentration (MFC). From the MIC and MFC data reported in Table 2, we could derive the following conclusions. The inactivity of FLCpOH confirmed that the drug derivative, used during the synthesis of our target compounds, did not exert any antifungal activity. All compounds displayed poor activity against the *C*. *glabrata* strain tested. The lower efficiency of azoles against *C. glabrata* and also *C. krusei* compared to *C. albicans* is well-documented in the literature. Our previous investigation revealed that 15 out of 81 strains of *C. glabrata* isolated from patients of three different hospitals in Poland were cross-resistant to fluconazole, voriconazole, posaconazole and itraconazole. Thirteen of them showed upregulation of the CDR1 gene encoding the efflux pump, which was the most probable reason for the low efficiency of the azoles [28]. The *C. glabrata* DSM6128 and *C. krusei* DSM11226 reference strains used for the study do not overproduce drug transporters. Other factors are responsible for the low activity of fluconazole; the combination of this agent with selected CPP did not result in a noticeable improvement of antifungal potential. The only (slightly) positive effect was noticed for *C. krusei*. Compounds FLCpOH-LFacinB[Nle^1,11^]-NH_2_, FLCpOH-LFacinB(2-11)-NH_2_, and FLCpOH-HLopt2-NH_2_ were found to be moderately or poorly active against the strains tested. Among all fluconazole-based conjugates, compounds FLCpOH-TP10-NH_2_ and FLCpOH-TP10-7-NH_2_ were found to be the most promising, particularly considering their activity against the *C. albicans* strains tested, including both reference strains and clinical isolates. The most important advantage of these two derivatives was high activity against fluconazole-resistant strains. Moreover, no important differences in susceptibility were observed for two pairs of clinical isolates, B3–B4 and Gu4–Gu5. Thus, both conjugates were not effectively removed from the cells of B4 and Gu5 strains that overproduce the drug pumps. In the case of fluconazole, differences in susceptibility of B3–B4 and Gu4–Gu5 were evident. Important differences were also observed between the resistance of B3 and B4 against FLCpOH-LFacinB[Nle^1,11^]-NH_2_, which suggests that this compound is a substrate of the MDR1 drug efflux pump (overproduced in B4). Two the most active agents identified in this step of the study, namely FLCpOH-TP10-NH_2_ and FLCpOH-TP10-7-NH_2,_ were selected for further investigation.

Another identified advantage of FLCpOH-TP10-NH_2_ and FLCpOH-TP10-7-NH_2_ is their higher fungicidal potential compared to fluconazole. The MFC values against *C. albicans* reference strains were the same or only twice higher compared to MIC_90_. The high fungicidal activity of both conjugates was also confirmed with the kill-time assay. However, at least a 4 × MFC concentration was necessary to obtain an evident killing effect after 24 h of treatment.

As mentioned in the introduction, none of the currently available antifungals used in clinical scenarios meet all expectations considering efficiency and safety. In this study, we used Amphotericin B as a control/reference. All strains exhibited a high level of susceptibility to this antibiotic. MIC values for all strains tested were below 0.1 µM. However, this agent is also well-known for its severe side effects, which include high fever, shaking chills, hypotension, nausea, vomiting, headache, dyspnea and tachypnea, but most importantly, kidney damage. Due to the high nephrotoxicity, AmB use is limited, treating only serious, life-threatening fungal infections. Other important disadvantages of AmB are its low solubility in water and high price. The performed assays revealed relatively low cytotoxicity of the conjugates synthesized herein, which also exhibited high fungicidal potential. Azoles are significantly cheaper than AmB; we can assume that production on a large scale of conjugates of azoles with CPP or AP would also be relatively inexpensive.

It is worth noting that in our previous results with other series of FLC conjugates, a peptidic component was attached not to the drug’s hydroxyl group, as in the case of the conjugates discussed here, but to its triazole ring. Such conjugates of FLC with LFcinB peptides displayed only marginal antifungal activity, demonstrating that the antibacterial activity, in this case, resulted rather from the intrinsic activity of the AMPs themselves [29]. Similarly, the coupling of TP10-NH_2_ to the FLC triazole ring (FLC-CH_2_CO-TP10-NH_2_, Figure S3 and Table S1 in Supplementary Material) yielding a practically inactive conjugate. The obtained results indicate that the substitution of FLC at the triazole ring causes a significant decrease in its antifungal activity, and no beneficial effect from the conjugation with the peptide was observed.

### 2.3. Time Kill Assay

**Figure 2:**
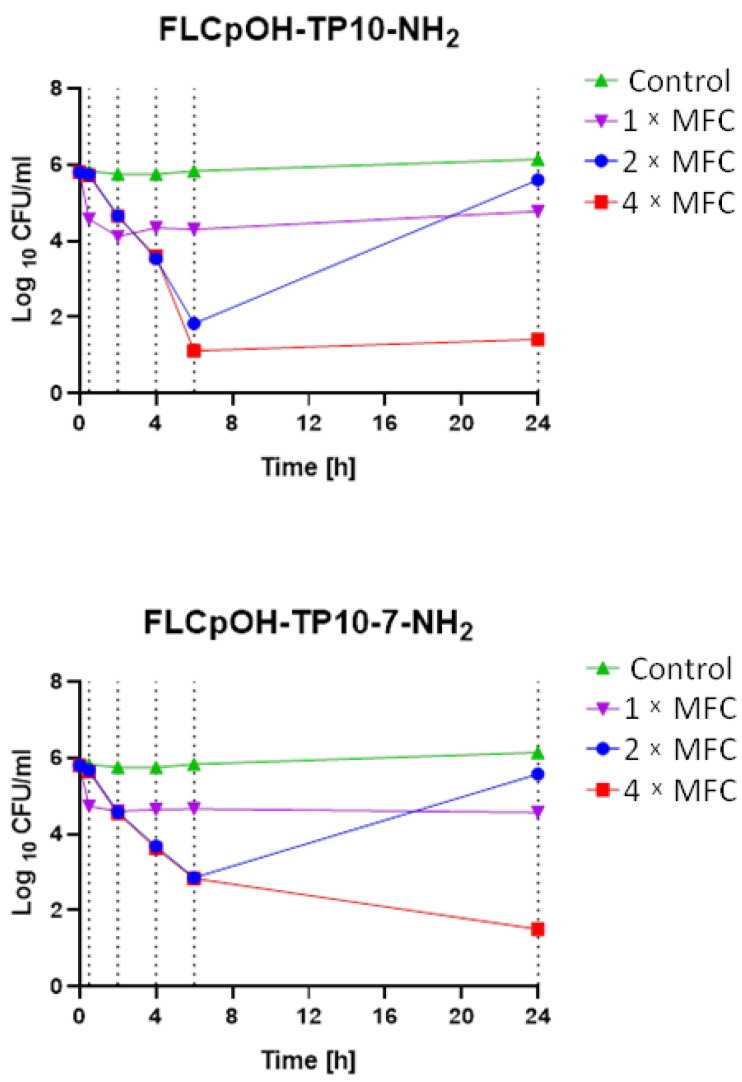
Time-kill determinations for *C. albicans* strain after treatment with FLCpOH-TP-NH_2_ and FLCpOH-TP10-7-NH_2_ alone at 1 × MFC, 2 × MFC and 4 × MFC. The *x*-axis represents the killing time, and the y-axis represents the logarithmic *C. albicans* SC5314 survival.

To determine the fungistatic or fungicidal nature of the compounds generated, time-kill assays over a 24 h period with two of the most active FLC derivatives (FLCpOH-TP10-NH_2_ and FLC-TP10-7-NH_2_) against *C. albicans* SC5314 were performed (Figure 2). At a concentration equal to 1 × MFC, both agents elicited only moderate fungicidal effect—1 log10 reduction in yeast cell count. Much more effective was the treatment at concentration of 2 × MFC. An important decrease in the number of yeast cells surviving in the suspensions was observed after 2, 4 and 6 h. However, this concentration was still not efficient enough, and after 24 h of incubation, the number of living cells in suspensions treated with both agents were only slightly lower compared to the control. Interestingly, even at the concentration of 4 × MFC, after 24 h the residual populations of living cells (between 10 and 100 CFU/mL) were still present in the suspensions treated with each conjugate. However, this result suggests a fungicidal rather than fungistatic (that is confirmed for fluconazole) mode of action of the produced conjugates.

### 2.4. Membrane Permeabilization Assay

**Figure 3:**
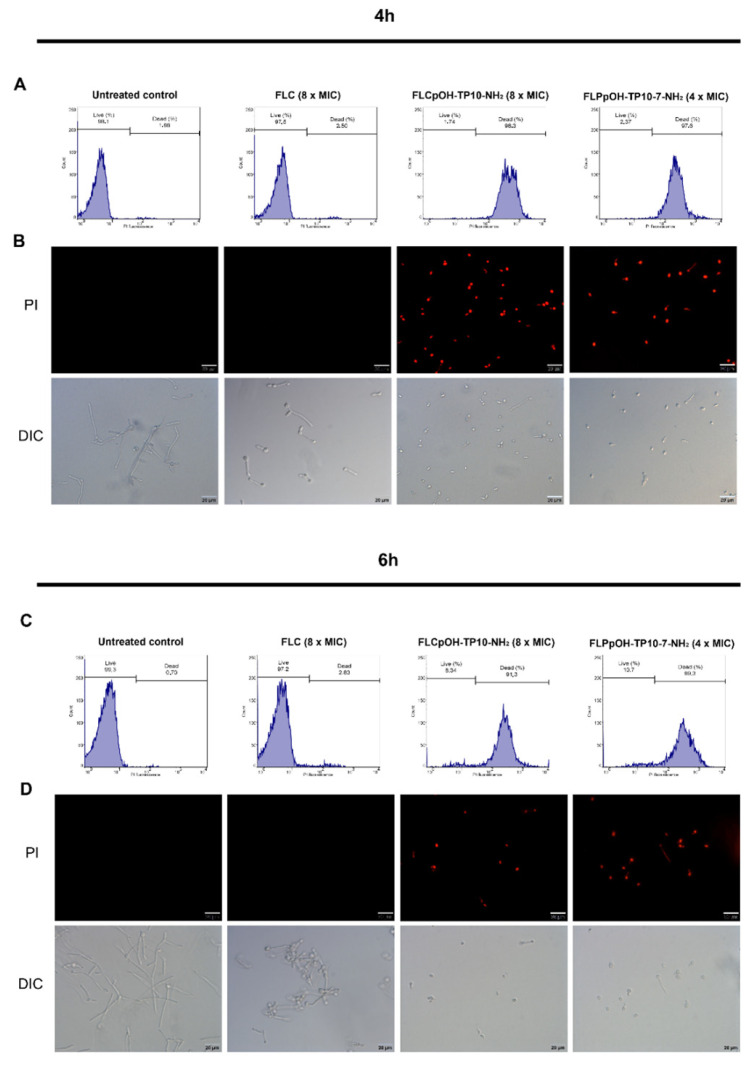
(**A**,**C**) Flow-cytometric analysis of membrane permeabilization assay by PI uptake. Cells were treated with FLC and FLC conjugates and stained with PI. After the completion of treatment and staining process, the cellular fluorescence was then analyzed via flow cytometry. (**B**,**D**) Fluorescence microscopy analysis of membrane permeabilization assay by PI uptake in treated as well as untreated yeast cells. The results of selected images are chosen as the best representatives of one of three different experiments with two replicates; similar results were observed each time. DIC—differential interference contrast microscopy, PI—fluorescence microscopy. Scale bars correspond to 20 µm.

The effect of synthesized compounds FLCpOH-TP10-NH_2_ and FLC-TP10-7-NH_2_ on fungal cell membrane integrity was evaluated with propidium iodide (PI) dye as a probe. Fluorescence microscopy and flow cytometry were used for this purpose. Alteration in membrane permeability is associated with a change in the physical state of the membrane or could result from compromised cell wall integrity. The dye can only enter cells with compromised membrane and binds to DNA by intercalating between the bases, with little or no sequence preference. As presented in Figure 3, important differences in the effect of treatment of *C. albicans* SC5314 with fluconazole and its derivatives were observed. No symptoms of cell membrane disruption were noticed in the case of yeast cells treated with fluconazole for 4 or 6 h at a concentration equal to 8 × MIC (62 µM). However, exposing the reference strain of *C. albicans* to FLCpOH-TP10-NH_2_ at a concentration of 8 × MIC (62 µM) and FLCpOH-TP10-7-NH_2_ at a concentration of 4 × MIC (60 µM) resulted in significant cell membrane damage that allowed the dye (PI) to enter the cells and bind with chromosomal DNA. This caused increased fluorescence of the cells in comparison with the untreated growth controls (or cells treated with fluconazole), which was measured with flow cytometry. The cells with disrupted cell membrane appeared in fluorescence microscopy as reddish cells, and substantial differences were observed between the cells treated with fluconazole and its derivatives. These results clearly confirmed other mechanisms of antifungal activity of fluconazole and conjugates of this agent with the selected TP10 peptides. As already mentioned, these peptides are classified as cell-penetrating peptides. It has been reported that TP10 can transport cargo across cell membranes. In addition, it can penetrate both mammalian and microbial cells. TP10 peptides preferentially permeabilized and killed microbes, but cytotoxicity against mammalian cells was also reported [29]. In this respect, TP peptides behave like AMPs, disrupting cell membranes. Structure–activity studies on TP10 led Xie et al. [27] to select an analogue named TP10-7 with reduced toxicity. Unfortunately, under the experimental conditions described above, the influence of both TP10-NH_2_ and TP10-7-NH_2_ on membrane cell disruption was similar.

### 2.5. SEM Analysis of the Compounds’ Effects on C. albicans Cells

**Figure 4:**
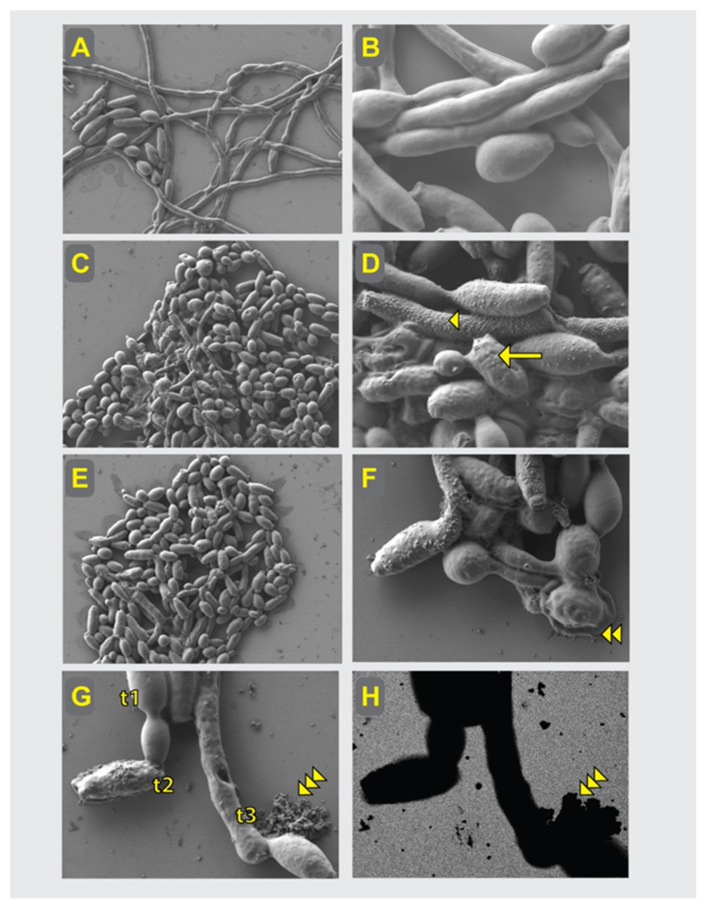
SEM analysis of the compounds’ effects on the *C. albicans* cells. Control cells (**A**,**B**) demonstrating normal appearance of *C. albicans*, with long hyphae stage well-represented (**A**) and uniformly smooth surface (**B**). Compound FLC-TP10-7-NH_2_, (**C**,**D**), in contrast, resulted in reduction of hyphae numbers (**C**), roughed cells surface (arrow) in large part of cell population, granularities (arrowhead), perforations and increased debris (**D**). Compound FLCpOH-TP10-NH_2_, (**E**–**H**) demonstrated signs of stronger toxicity against *C. albicans*, lack of hyphae (**E**), with intense roughing of the cell surface and membrane disintegration or spreading (double arrowheads) (**F**), frequent perforations and granularities (**G**), membrane blebbing and more debris. (**G**,**H**) Different stages of deleterious effects of the compound, from mild (t1), through roughing (t2) to perforation and fragmentation of cells (t3). Chemical contrast imaging (**H**), correlated pixel-to-pixel with topography imaging. (**G**) Improved identification of blebbing and fragmentation of *C. albicans* cells (triple arrowheads). Gray appearance of the substrate in (**H**) stems from silicon polished crystal and facilitates detection of multiple organic fragments with dark appearance in (**H**), by chemical contrast mapping of non-coated sample. (**A**–**G**)—Everhart–Thornley detector, (**H**)—EsB detector imaging. (**A**,**C**,**E**)—magnification 5000× (field of view 60 μm), (**B**,**D**,**G**,**H**)—magnification 20,000× (field of view 15 μm).

The *C. albicans* cells were analyzed at several time-points of live culture conditions with optimal compound concentrations chosen arbitrarily based on the above-described pharmacological profiling. The SEM analysis presented here describes drug effects upon 24 h of exposure to *Candida albicans.* Images of *C. albicans* cultured in phosphate-buffered saline (PBS) were chosen to ensure chemically distinguishing native organic carbon (dark appearance in EsB detector) from inorganic salt artifacts (bright appearance) that sometime appeared in the fields of view. Control culture without the tested compounds demonstrated *C. albicans* single cells and hyphae, both with smooth surface and no toxic effects (Figure 4A,B; magnification 5000× and 20,000×, respectively). Control-group cell populations did not demonstrate fragmentation or vesicular fragments of fungal organisms. In contrast, the effects of compound FLC-TP10-7-NH_2_ at a concentration of 4 × MIC could be noticed as a dramatic reduction in the number of hyphae, deterioration of the whole cells visible as roughed cell surface with varying intensity of granularity, local perforations, vesicular fragments deriving from the cells and dead cell debris (Figure 4C,D magnification 5000× and 20,000×, respectively). The effects of compound FLCpOH-TP10-NH_2_, at a concentration of 8 × MIC were even further expressed (Figure 4E,F; magnification 5000× and 20,000×, respectively). In addition to signs of toxicity visible as roughing of the cell surface, perforations and granularities, membrane blebbing and more frequent debris of the dead cells could be identified. In Figure 4G,H we present the representative picture of the deleterious effects of the FLCpOH-TP10-NH_2_ compound with an array of mild-to-strong (upper-left to lower-right, respectively) phenotypes of *C. albicans’* response to the tested drug (magnification 30,000×). Panel G shows the high-resolution topography features, while panel H demonstrates their chemical contrast, both correlated at pixel-to-pixel accuracy, which together with G illustrates the organic matter forms (dark appearance) on the background of the silicon surface (gray appearance) of the deteriorating fungal cells, in a correlative mode at different stages of the pathogens’ death.

### 2.6. Cytotoxicity

The adverse drug effects associated with the use of antimicrobials can be a major concern especially with antifungal agents due to the eukaryotic nature of both the organism being targeted and the host [30]. Therefore, it was important to test the toxicity of novel fluconazole-based conjugates. To examine and compare the cytotoxicity of FLC and its derivatives, the cytotoxicities of the compounds were assessed by the 3-(4,5-dimethylthiazol-2-yl)-2,5-diphenyltetrazolium bromide (MTT) assay in two mammalian cell lines: human foreskin fibroblast cell line (Hs27) and human umbilical venous endothelial primary cells (HUVEC). The results showed that the FLC appeared to be less cytotoxic than its conjugates (Table 3). Among conjugates, FLCpOH-TP10-NH_2_ and FLCpOH-TP10-7-NH_2_ appeared to be more cytotoxic. However, the IC_90_ value for the human cells after 72 h treatment was comparable to the MIC_50_ value after 24 h treatment for most strains of *C. albicans* (Table S2).

## 3. Conclusions

The results of our study are mostly in line with results presented by other authors who also observed that hybridization of different biologically active agents with fluconazole (other azoles) can lead to potent antifungal drugs. Interesting properties were exhibited, e.g., by carbazole-triazole conjugates. One of the synthesized compounds, namely 3,6-dibromocarbazolyl triazole, displayed excellent inhibitory efficacy against most of the tested fungal strains (MIC = 2–32 μg/mL) and effective fungicidal ability towards *C. albicans*, *C. tropicalis* and *C. parapsilosis* (MFC = 4–8 μg/mL) [31]. Elias and coworkers (2019) [32] showed that hybrids of cumarin and antifungals could penetrate into endoplasmic reticulum of fungal cells, where true target CYP51 generally exists. This potentially can enhance the efficacy of azole antifungal drugs. Aneja and coworkers (2016) [33] synthesized triazole–amino acid hybrids with potent in vitro and in vivo inhibitory activity against different *Candida* species. Some of the produced derivatives, particularly compounds marked as 68 and 70, showed potent in vitro activity against fluconazole-resistant as well as -sensitive clinical isolates of *C. albicans*. The 1,2,4-triazole-indole hybrid molecule synthesized by Paginez and colleagues (2019) [34] showed broad-spectrum activity against *Candida* spp., particularly against low fluconazole-susceptible species—*C. glabrata* and *C. krusei*. High activity against non-albicans *Candida* spp. was shown also by piperazine–azole hybrids produced by Thamban Chandrika (2018) [35]. Han et al., (2020) [36] designed hybrids of (CYP51)-histone deacetylase (HDAC) dual inhibitors. The synthesized agents revealed high antifungal activity, including against azole-resistant strains. Antifungal mechanism studies revealed that they acted by blocking ergosterol biosynthesis and HDAC catalytic activity in the fungus, suppressing the function of the efflux pump, yeast cell-to-hypha morphological transition, and biofilm formation. Conjugates of fluconazole and flucytosine synthesized by Fang et al. (2017) [37] efficiently inhibited the growth of *C. albicans* ATCC 90023 as well as clinically resistant strain *C. albicans* with MIC values of 0.008 and 0.02 mM, respectively. Many of these conjugates showed fungicidal rather than fungistatic activities and affected more than one (lanosterol 14α-demethylase) molecular target within yeast cells. In conclusion, both data from the literature and the results of this study confirm that the design and synthesis of hybrids of azoles with a large variety of different compounds that exhibit antimicrobial activity is a promising approach for obtaining new, effective, and safer antifungals.

Additionally, it is worth stressing that to our knowledge, the use of the hydroxyl group of fluconazole for linking with the peptide has not been applied to obtain conjugates with FLC so far and has not been described in the literature.

## 4. Materials and Methods

### 4.1. Solid-Phase Peptide Synthesis (SPPS)

All peptides were synthesized at 50 μmole scale by the solid-phase protein technology approach using Fmoc/Boc chemistry with an automatic Prelude peptide synthesizer (Gyros Protein Technology, Inc., Tucson, AZ, USA), as described previously [22]. Peptides were synthesized on TentaGel S RAM resin (loading 0.24 meq/g, Rapp Polymere, Tuebingen, Germany) to obtain peptides with an amide group on their *C*-termini after cleavage. The peptide chain was elongated in the consecutive cycles of deprotection and coupling. Deprotection was performed with 20% piperidine in *N*,*N*-dimethylformamide (DMF), and peptide chain elongation was effected using three-fold molar excess of *N,N,N′,N′-*tetramethyl-O-(benzotriazol-1-yl)uroniumtetrafluoroborate (TBTU)/1-hydroxybenzotriazole (HOBt)/*N*-methylmorpholine (NMM) and *N*-α-Fmoc and side-chain protected amino acid (GL Biochem, Shanghai, China). After completing the synthesis, peptides and peptide conjugates were cleaved from the resin, and the protecting groups were removed in a one-step procedure using a mixture of TFA:phenol:triisopropylsilane:H_2_O (88:5:2:5, *v*/*v*/*v*/*v*). The crude conjugates were purified on a Beckman Gold System (Beckman, USA) equipped with RP Supelco Discovery BIO, Wide Pore C8, 10 mm column (10 × 250 mm, Sigma Aldrich, Darmstadt, Germany). The solvent systems were 0.1% TFA in water (A) and 0.1% TFA in 80% acetonitrile in water (B). Different linear gradients were applied (flow rate 5.6 mL min^−1^, monitored at 226 nm). The purities of the synthesized conjugates were checked with an HPLC Pro Star system (Varian, Mulgrave, Australia) using a Kinetex 5 μm XB-C18 100 Å column (4.6 × 150 mm, Phenomenex^®^, Torrance, CA, USA). The solvent system was used as described above. Linear gradient from 10 to 90% B for 40 min, flow rate 1 mL min^−1^, monitored at 226 nm was used. All described compounds were of at least 95% purity. In order to confirm the correctness of the molecular masses of the synthesized peptides and peptide conjugates, mass spectrometry analysis was carried out by MALDI-TOF MS (Biflex III MALDI-TOF spectrometer, Bruker Daltonics, Ettlingen, Germany or MALDI TOF/TOF 5800+ spectrometer, AB SCIEX, Framingham, Massachusetts, USA) with an α-cyano-4-hydroxycinnamic acid (CCA) and/or 2,5-dihydroxybenzoic acid (DHB) matrix.

### 4.2. Synthesis of FLCpOH

**Figure 5:**
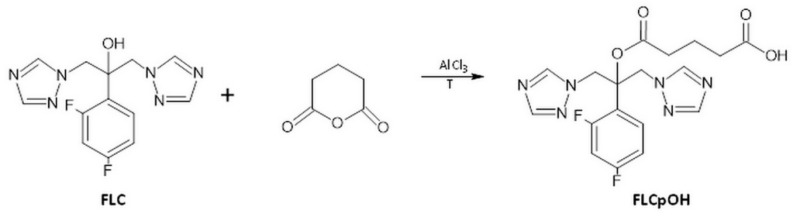
Scheme of the synthesis of fluconazole derivative (FLCpOH) (reaction conditions: AlCl_3_, 120 °C, 45 min).

Glutaric anhydride (570 mg, 5 mmol) and fluconazole (306 mg, 1 mmol catalytic amount of AlCl_3_) were placed in a microwave reactor (Plazmatronika RM800, Ertec Inc., Poland) at a power of 120 W and a temperature of 120 °C for approximately 45 min (Figure 5). Then, the reaction mixture was cooled to room temperature, dissolved in 10–15 mL of the solvent mixture water:acetonitrile (8:2, *v*/*v*), and solid residue was filtered off. The crude product was purified on a Beckman Gold System (Beckman, Indianapolis, USA) equipped with RP Supelco Discovery BIO, Wide Pore C8, 10 mm column (10 × 250 mm, Sigma Aldrich, Darmstadt, Germany). The solvent systems were 0.1% TFA in water (A) and 0.1% TFA in 80% acetonitrile in water (B). Linear gradient from 15 to 30% B for 25 min, flow rate 5.6 mL min^−1^, monitored at 226 nm was used; yield 118 mg, 38%. The product was confirmed by HPLC, MALDI-TOF and NMR.

^1^ H NMR and ^13^C NMR experiments were performed on a 5 μm XB-C18 100 Å column (4.6 × 150 mm, Phenomenex^®^, Torrance, CA, USA). The solvent system was used as described above. Linear gradient from 10 to 90% B for 40 min, flow rate 1 mL min^−1^, monitored at 226 nm was used. All described compounds were of at least 95% purity. In order to confirm the correctness of molecular masses of the synthesized peptides and peptide conjugates, mass spectrometry analysis was carried out by MALDI-TOF MS (Biflex III MALDI-TOF spectrometer, Bruker Daltonics, Ettlingen, Germany or MALDI TOF/TOF 5800+ spectrometer, AB SCIEX, Framingham, Massachusetts, USA) with an α-cyano-4-hydroxycinnamic acid (CCA) and/or 2,5-dihydroxybenzoic acid (DHB) matrix.

^1^H NMR and ^13^C NMR experiments were performed on a Bruker Avance III 700 MHz. TSP (10 mM) was used as an internal standard. NMR spectra (Supplementary Material, Figure S2) were recorded for 26 mM solution in DMSO (0.5 mL) of the analyzed compound FLCpOH. The analysis results are given bellow:

^1^H NMR (500 MHz, DMSO): δ = 8.31 (s, 2H), 7.94 (s, 2H), 7.26–7.20 (m, 1H), 7.08–7.02 (m, 1H), 6.99–6.93 (m, 1H), 5.17 (d, J = 14.8 Hz, 2H), 5.02 (d, J = 14.8 Hz, 2H), 2.39 (t, J = 7.4 Hz, 2H), 2.28 (t, J = 7.3 Hz, 2H), 1.76–1.69 (m, 2H); ^13^C NMR (126 MHz, DMSO): δ = 174.0, 171.4, 163.2, 163.1, 161.2, 161.1, 160.0, 159.9, 158.0, 157.9, 151.5, 145.6, 128.9, 120.5, 120.4, 111.6, 111.5, 104.9, 104.8, 104.5, 80.7, 51.2, 33.3, 32.5, 19.4.

### 4.3. Synthesis of Fluconazole-Based Conjugates

The mixture of purified FLCpOH, TBTU, and DIPEA (in molar ratio 1:1:2) was dissolved in 8 mL of DMF:DCM (1/1; *v*/*v*), added to SPPS vessel with peptidyl resins with free *N*-terminal amino groups. The coupling efficiency was monitored every 12 h using the chloranil test. In case of a positive test result, acylation was repeated. Usually, 24–48 h was required to complete the coupling. The conjugates were cleaved from the resin as described above. The crude products were purified and analyzed as described above; the yields were 45–56%.

### 4.4. Antifungal Agents

Fluconazole derivatives were chemically synthesized as described above. A 10 µM stock solution of compounds was prepared in DMSO and stored at 4 °C. The antifungal agents, fluconazole (FLC) and Amphotericin B (AmB), were purchased from Sigma-Aldrich, Poznan, Poland. Both of these antifungals were also dissolved in DMSO at final concentrations of 10 µM and stored at 4 °C.

### 4.5. Organisms and Culture Conditions

The assessment of antifungal potential of synthesized agents was carried out using four reference strains (*C. albicans* ATCC 10231, *C. albicans* SC5314, *C. glabrata* DSM 11226 and *C. krusei* DSM 11226) and six clinical isolates of fluconazole-resistant *C. albicans* that were provided by the Hospital of Medical University of Gdansk, Poland. Moreover, four *C. albicans* isolates, assigned as B3, B4, Gu4 and Gu5, were used for investigation whether the synthesized fluconazole derivatives were effectively removed from the yeast cells by the drug efflux transporters, as in the case of fluconazole. These strains were kindly provided by Prof. Joachim Morschhäuser from University of Würzburg (Würzburg, Germany). Strains Gu4 and B3 were fluconazole-sensitive isolates obtained from early infection episodes, while Gu5 and B4 were the corresponding fluconazole-resistant isolates obtained from later episodes in the same patients treated with fluconazole. The lack of susceptibility of the Gu5 strain to fluconazole was a consequence of overexpression of CDR1/2 genes encoding ABC transporters, whereas the resistance of B4 strain was caused by overexpression of the MDR1 gene encoding a membrane transport protein of the major facilitator superfamily (MFS) [38]. All of these *Candida* spp. strains were maintained on YPD agar plates (1% (*w*/*v*) yeast extract, 2% (*w*/*v*) peptone, 2% (*w*/*v*) glucose, 2% (*w*/*v*) agar) at 30 °C for 16–24 h. For all of the assays aimed at assessing the antifungal potential of the synthesized agents, the yeasts were cultivated at 37 °C in RPMI 1640 medium (with L-glutamine, without sodium bicarbonate, Sigma-Aldrich, Darmstadt, Germany)) buffered to a pH of 7.0 with 0.165 M morpholinepropanesulfonic acid (MOPS) buffer (Sigma-Aldrich). In this study, we used the human foreskin fibroblast cell line Hs27 (ATCC CRL 1634TM) and the human endothelial cell line HUVEC (C2519A), purchased from Lonza Group AG, (Basel, Switzerland). The Hs27 cells were maintained in cell media consisting of Dulbecco’s modified Eagles medium (DMEM), (Corning, Arizona, USA) with 10% fetal bovine serum (FBS), (Corning, Port Elizabeth, South Africa), 1% penicillin/streptomycin (Sigma-Aldrich Inc., Darmstadt, Germany), and 1% L-glutamine (Corning, Arizona, USA). The human endothelial cell line was maintained in cell media consisting of Endothelial Cell Growth Medium-2 Single Quots Kit (Lonza, USA). Both cell lines were cultured in an incubator at 37 °C and 5% CO_2_.

### 4.6. Determination of Minimum Inhibitory Concentration (MIC) and Minimum Fungicidal Concentration (MFC)

Antifungal activity of the tested compounds against yeast cells was determined in 96-well plates as described in the CLSI document M27-A3 [39], with minor modifications (CLSI, 2008). Suspensions of the microorganisms were prepared by taking one loop of pure culture into sterile water and adjusting optical density to 0.1 at 660 nm wavelength and further 50-fold dilution in RPMI 1640 medium, resulting in cell concentration of approximately 2 × 104 CFU/mL [21]. Then, 100 μL of cells were added to the wells of a 96-well microtiter plate that contained 16–500 μM tested compounds and 16–500 μM of FLC or 0.2–62 μM AmB. The plates were incubated for 24 h at 37 °C. Each test was performed in triplicate. The final concentration of DMSO was ensured to be around 1% in all experiments. The MIC_90_ was defined as the lowest concentration of drug showing no visible growth; MIC_50_ was defined as the concentration that yielded at least 50% growth inhibition when compared with the growth control well.

For determination of minimum fungicidal concentrations (MFC), small aliquots of suspensions (around 10 μL) from each well were transferred using the pipette to YPD agar plates without inhibitors and incubated for 24 h at 37 °C. The MFC was defined as the lowest concentration of drug at which no growth of the colonies was observed.

### 4.7. Time-Kill Assay

Antifungal time-kill studies were carried out by a method previously described and evaluated by Klepser et al. [40]. A starting inoculum was prepared by inoculating a tube of sterile water with growth from 24 h YPD agar plates (*C. albicans* SC5314), and OD660 was adjusted to about 0.1 (around 106 cells per 1 mL). The suspension was then diluted 10-fold with RPMI 1640 medium, and FLCpOH-TP10-NH_2_ and FLCpOH-TP10-7-NH_2_ were added at concentrations corresponding to 1 × MFC, 2 × MFC, and 4 × MFC. A negative control (growth medium without drug or organism) was also included at each time of testing. Tubes were incubated at 37 °C on an orbital shaker and vortexed before removing a sample for the determination of colony counts. After the appropriate time of incubation (0.5, 2, 4, 6, and 24 h), 1 mL of each suspension was centrifuged (2 min, 9170× *g*), and the pellet was re-suspended in 1 mL of PBS pH 7.4. Ten-fold serial dilutions with PBS were prepared, and 100 μL of each was inoculated on YPD agar plates in duplicate. Plates were incubated for 24 h at 37 °C. Colony forming units in the range of 30–300 were counted (on the plates containing from 30 to 300 colonies) and the number of yeast cells in 1 mL (CFU/mL) was calculated [21].

### 4.8. Membrane Permeabilization Assay Using Propidium Iodide Staining

Suspensions of *C. albicans* SC5314 were prepared by taking one loop of pure culture growth from 24 h YPD agar plates into RPMI 1640 medium and adjusting optical density to 0.1 at 660 nm wavelength. Then, FLCpOH-TP10-NH2 and FLCpOH-TP10-7-NH2 were added at concentrations corresponding to 8 × MIC and 4 × MIC. FLC (8 × MIC) was used as a negative control. The cell suspensions were then treated for 0.5, 2, 4, 6, and 24 h at 37 °C with continuous agitation (180 rpm). The cells were then centrifuged and resuspended in 200 µL of PBS buffer (pH 7.4). Subsequently, cells were treated with propidium iodide (1 mg/mL, final concentration) and incubated for 30 min at room temperature in the dark. Cellular fluorescence was visualized using lens 20×, using Olympus Fluorescence Microscope BX60 (Olympus, Tokyo, Japan). The images were also post-processed utilizing cell Sens program. The fluorescence intensity emitted by DNA-bound propidium iodide was measured using a flow cytometer (Merck Millipore guava easyCyte 8, Darmstadt, Germany). Data were obtained from at least two independent experiments.

### 4.9. Scanning Electron Microscopy (SEM)

Scanning electron microscopy of yeast was performed at a low accelerating voltage of the primary beam with or without the coating of the samples, as described in previous work [41]. *C. albicans* fungi suspension fixed with 2.5% glutaraldehyde was applied onto a silicon chip and allowed to adhere for 30 min. The samples were dehydrated in a series of methanol solutions (25–50–75–100–100%) in one-hour steps at 4 °C. Samples underwent critical point drying with methanol exchanged for liquid CO_2_ in an automatized approach, (CPD300 AUTO, Leica Microsystems, Vienna, Austria) and were imaged with cross-beam scanning electron microscope equipped with Schottky field-emission cathode (Auriga 60, Carl Zeiss, Oberkochen, Germany) at 0.8 kV accelerating voltage; thus, the imaging was performed within a mode referred to as low-voltage, field-emission scanning electron microscopy (LV-FESEM) of non-labeled, critical point-dried sample. This process was implemented with the low-voltage field-emission scanning electron microscopy (LV-FESEM) mode and by applying the low-energy loss electron principle for generating the highly resolved chemical contrast, as described in the review paper of Drab (2018) [41]. Images were acquired with the Everhart–Thornley electron detector (SE2 secondary electrons) and correlated with the energy-selective back-scattered electron detector (EsB), directly from the sample surfaces, with no coating or contrasting applied; thus, chemical cell-endogenous components were directly mapped [42]. Polished silicon crystal used as a substrate for deposition of *C. albicans* allowed for chemical mapping of the sample’s features and for identifying of the disintegrated pathogen cells in a correlative mode, with topography, contours and chemical aspects analyzed with pixel-to-pixel precision of correlation [41].

### 4.10. Cytotoxicity Assay

Hs27 and HUVEC mammalian cell lines were cultured as mentioned above. Once cell lines were about 80% confluent, cells were counted by a hemacytometer (Hausser Scientific, Horsham, PA, USA) and plated in 96-well microtiter plates at concentrations of 4500 cells per well for Hs27 and 2000 cells per well for HUVEC. Cells were allowed to attach overnight. Drugs were serial dissolved (100–1.5 μM) in cell culture medium and added to wells in 100 µL, in triplicates. The final concentration of DMSO was ensured to be around 1% in all experiments. Cells were incubated with studied compounds for 72 h at 37 °C and 5% CO_2_. After incubation, 20 µL of MTT solution in PBS (4 mg/mL) was added to all wells and incubated further for 3 h at 37 °C. Formed formazan crystals were dissolved in 150 µL DMSO, and absorbance was measured using an Asys UVM340 multi-well plate reader at λ = 540 nm. Cytotoxicity was determined compared to drug-free control. All experiments were performed in biological triplicates.

## Figures and Tables

**Table 1:** Physicochemical properties of FLCpOH, the peptide conjugates and their components.

Compound	Calculated Monoisotopic Molecular Weight	Measured Accurate Mass [M + H]^+^ [*m*/*z*]	t_R_ (min)	Yield %
FLCpOH	420.2	421.1	16.5	38%
LFcinB(2-11)-NH_2_	1494.8	1495.8	14.3	95%
LFacinB[Nle^1,11^]-NH_2_	2016.2	2017.6	17.4	95%
TP10-7-NH_2_	2278.5	2278.5	22.4	90%
TP10-NH_2_	2180.4	2181.4	27.0	80%
HLopt2-NH_2_	1606.9	1607.9	12.4	95%
FLCpOH-LFacinB(2-11)-NH_2_	1896.8	1897.9	19.5	56%
FLCpOH-LFacinB[Nle^1,11^]-NH_2_	2418.4	2419.8	20.8	53%
FLCpOH-TP10-7-NH_2_	2679.5	2680.8	27.8	45%
FLCpOH-TP10-NH_2_	2582.6	2583.3	29.5.	50%
FLCpOH-HLopt2-NH_2_	2008.9	2010.0	17.5	49%

**Table 2:** MIC and MFC Values (in µM) Determined for Compounds, As Well As for Two Control Antifungal Agents (AmB and FLC) against Various Yeast Strains.

Yeast Strains	MIC * and MFC ** [µM]	Compounds							
		FLCpOH	FLCpOH-TP10-NH_2_	FLCpOH-TP10-7-NH_2_	FLCpOH-LFacinB[Nle^1,11^]-NH_2_	FLCpOH-LFacinB(2-11)-NH_2_	FLCpOH-HLopt2-NH_2_	Fluconazole (FLC)	Amphotericin B (AmpB)
*Candida glabrata* DSM 6128	MIC_50_	>250	62	190	250	>250	>250	62	<0.1
	MIC_90_	>250	125	250	>250	>250	>250	250	<0.1
	MFC	-	-	-	-	-	-	-	<0.1
*Candida krusei* DSM 11226	MIC_50_	>250	62	31	31	31	>250	70	<0.1
	MIC_90_	>250	125	62	62	62	>250	125	<0.1
	MFC	-	250	62	-	125	-	-	<0.1
*Candida albicans* SC 5314	MIC_50_	>250	8	8	23	62	62	<8	<0.1
	MIC_90_	>250	15	15	31	125	125	<8	<0.1
	MFC	-	31	15	250	250	-	-	<0.1
*Candida albicans* ATCC 10231	MIC_50_	>250	15	15	45	125	125	<8	<0.1
	MIC_90_	>250	31	31	62	250	250	<8	<0.1
	MFC	-	62	62	-	-	-	-	<0.1
*Candida albicans* B3	MIC_50_	>250	31	31	45	125	>250	-	<0.1
	MIC_90_	>250	62	62	62	250	>250	15	<0.1
	MFC	-	62	62	-	-	-	-	<0.1
*Candida albicans* B4	MIC_50_	>250	62	45	250	190	>250	-	<0.1
	MIC_90_	>250	125	62	>250	250	>250	62	<0.1
	MFC	-	125	62	-	-	-	-	<0.1
*Candida albicans* Gu4	MIC_50_	>250	45	23	125	>250	>250	-	<0.1
	MIC_90_	>250	62	31	250	>250	>250	31	<0.1
	MFC	-	62	31	-	-	-	-	<0.1
*Candida albicans* Gu5	MIC_50_	>250	62	31	>250	>250	>250	-	<0.1
	MIC_90_	>250	125	62	>250	>250	>250	>250	<0.1
	MFC	-	62	62	-	-	-	-	<0.1
*Candida albicans* 48	MIC_50_	>250	45	15	250	250	250	-	<0.1
	MIC_90_	>250	62	31	>250	>250	>250	>250	<0.1
	MFC	-	250	125	-	-	-	-	<0.1
*Candida albicans* 138	MIC_50_	>250	45	23	23	190	>250	-	<0.1
	MIC_90_	>250	62	31	31	250	>250	>250	<0.1
	MFC	-	250	125	-	-	-	-	<0.1
*Candida albicans* 190	MIC_50_	>250	45	23	90	190	250	-	<0.1
	MIC_90_	>250	62	31	125	250	>250	>250	<0.1
	MFC	-	250	125	-	-	-	-	<0.1
*Candida albicans* 247	MIC_50_	>250	45	23	45	190	>250	-	<0.1
	MIC_90_	>250	62	31	62	250	>250	>250	<0.1
	MFC	-	250	125	-	-	-	-	<0.1
*Candida albicans* 574	MIC_50_	>250	45	23	45	190	250	-	<0.1
	MIC_90_	>250	62	31	62	250	>250	>250	<0.1
	MFC	-	-	250	-	-	-	-	<0.1
*Candida albicans* 604	MIC_50_	>250	45	23	45	190	>250	-	<0.1
	MIC_90_	>250	62	31	90	250	>250	>250	<0.1
	MFC	-	250	250	-	-	-	-	<0.1

* MIC—minimum inhibitory concentration**;** ** MFC—minimum fungicidal concentration.

**Table 3:** In vitro cytotoxicity (IC_50_ and IC_90_ ± SEM (μM)) of the FLC conjugates and their constituents toward Hs27 and HUVEC cells.

Compound	Hs27		HUVEC	
	IC_50_	IC_90_	IC_50_	IC_90_
FLC	>100	>100	>100	>100
FLCpOH	>100	>100	>100	>100
FLCpOH-TP10-NH_2_	12.00 ± 0.93	28.81 ± 0.87	14.45 ± 1.13	34.30 ± 1.00
FLCpOH-TP10-7-NH_2_	13.07 ± 0.35	29.78 ± 0.29	15.16 ± 1.26	34.03 ± 1.90
FLCpOH-LFacinB[Nle^1,11^]-NH_2_	>100	>100	75.58 ± 1.73	>100
FLCpOH-LFacinB(2-11)-NH_2_	>100	>100	>100	>100
FLCpOH-HLopt2-NH_2_	>100	>100	>100	>100

## Data Availability

Not applicable.

## Supplementary Materials

The following supporting information can be downloaded at: https://www.mdpi.com/article/10.3390/pharmaceutics14040693/s1, Figure S1. HPLC chromatograms (A) and MS analysis (B) of the peptide conjugates and their constituents; Figure S2. NMR spectra of FLCpOH (A), ^1^H NMR (B) and ^13^C NMR (C); Figure S3. The chemical structure of fluconazole derivative (FLC-COOH) and its conjugate with TP10-NH_2_; Table S1. The antifungal activity of peptides and conjugates in RPMI (200–0.4 μg/mL range); Table S2. Selectivity indexes (SI) of the FLCpOH-TP10-NH_2_ and FLCpOH-TP10-7-NH_2_ compounds. The selective activities of the FLC-conjugates were calculated according to the following formula “Selectivity index (SI) = (IC_90_ in µM after 72h) / (MIC_50_ in µM after 24h).
