# Supplementary material for: New Peptide Based Fluconazole Conjugates with Expanded Molecular Targets
Source: Pharmaceutics. 2022 Mar 23;14(4):693. doi: 10.3390/pharmaceutics14040693 (PMC9026428; doi:10.3390/pharmaceutics14040693)
Supplement: Supplementary file 1 [file pharmaceutics-14-00693-s001.zip › pharmaceutics-1612841-supplementary.pdf]

pharmaceutics

MDPI

Supplementary Materials: New Peptide Based Fluconazole Conjugates with Expanded Molecular Targets

Wioletta Brankiewicz, Joanna Okońska, Katarzyna Serbakowska, Jan Lica, Marek Drab, Natalia Ptaszyńska, Anna Łęgowska, Krzysztof Rolka and Piotr Szweda

FLCpOH

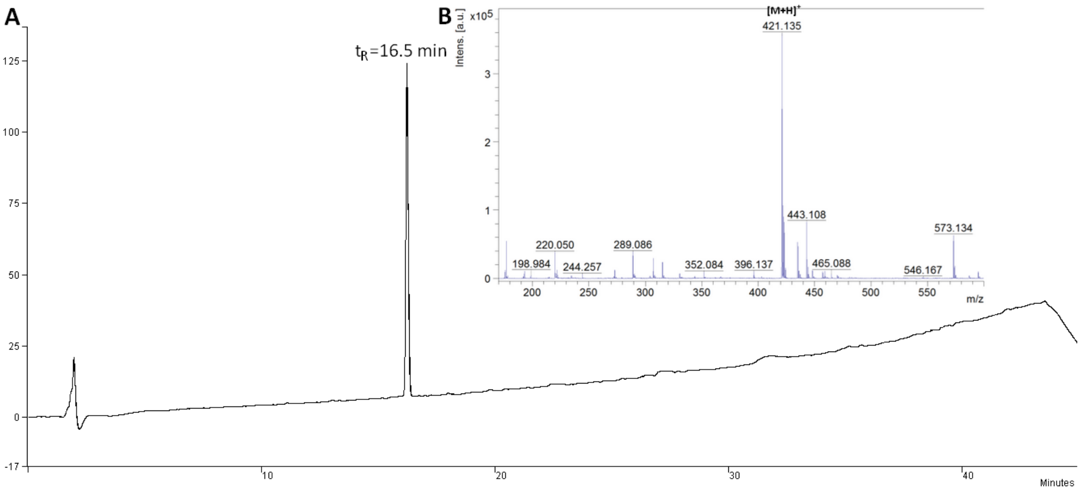

LFcinB(2-11)-NH$_2$

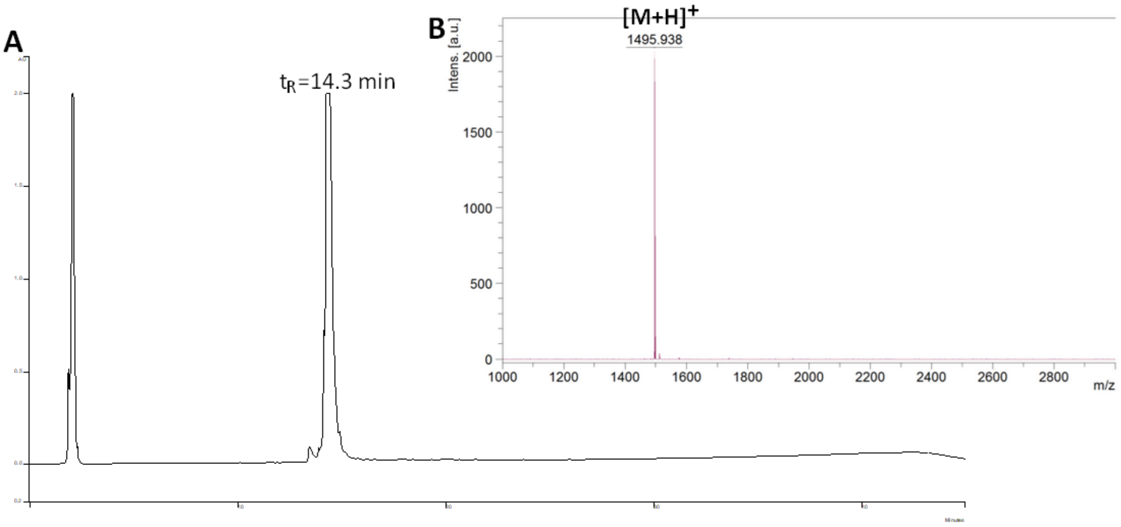

LFacinB[Nle^{1,11}]-NH$_2$

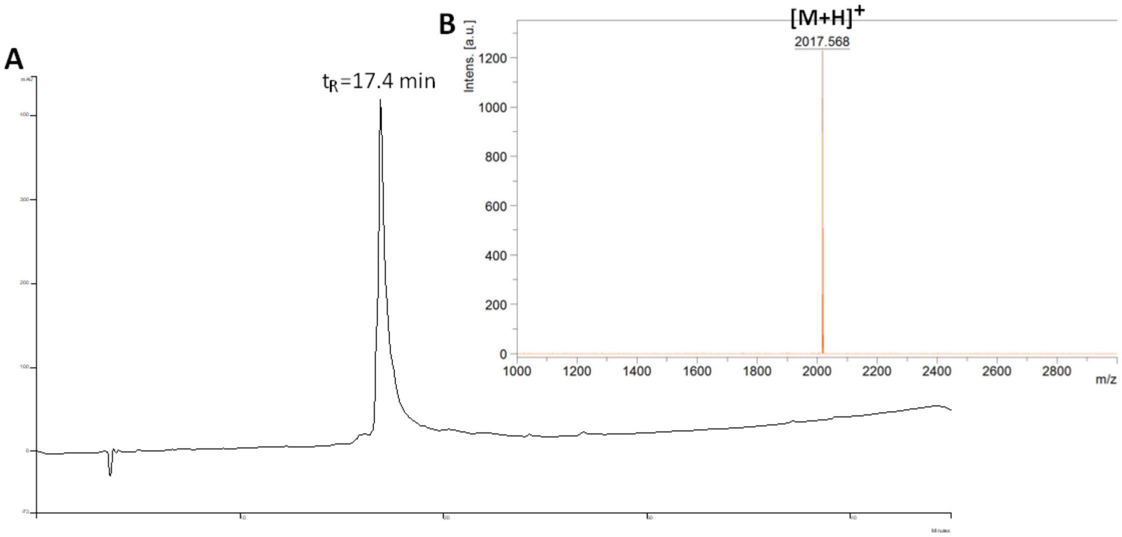

TP10-7-NH_2

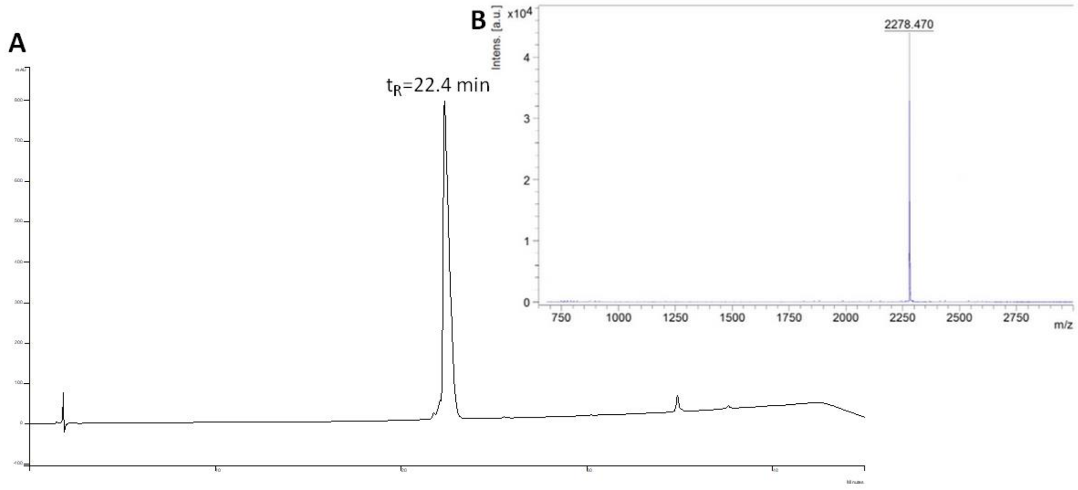

TP10-NH_2

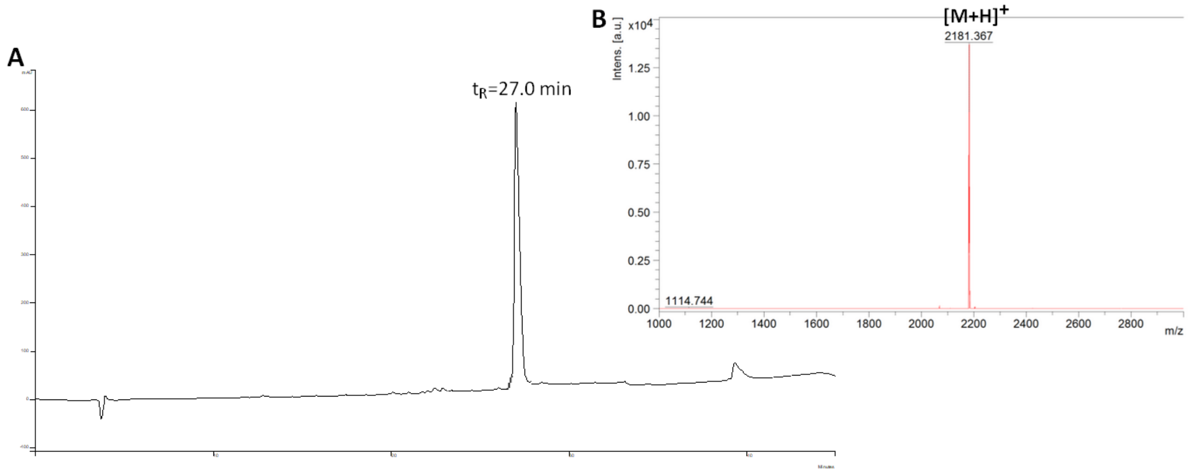

HLopt2-NH_2

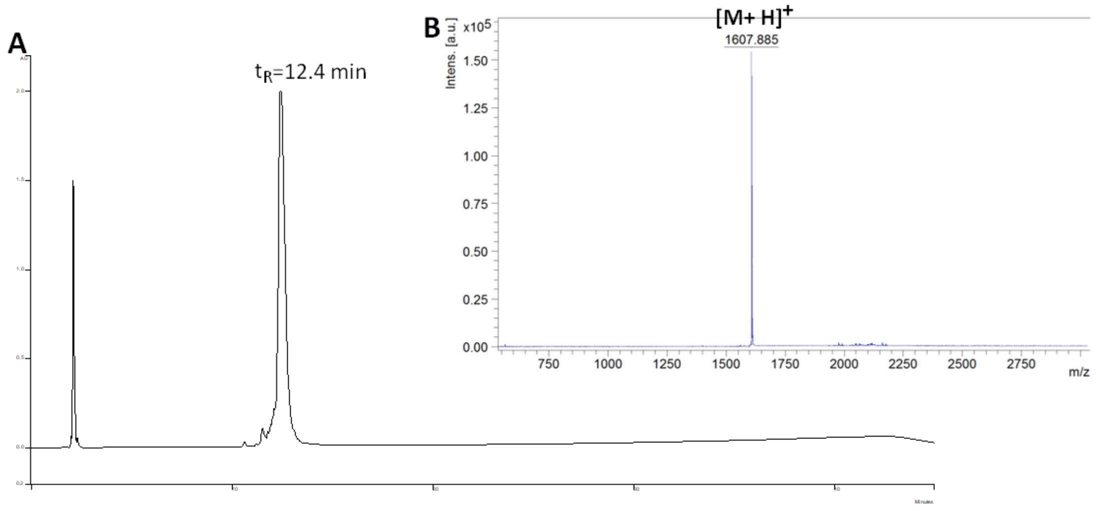

FLCpOH-LFacinB(2-11)-NH_2

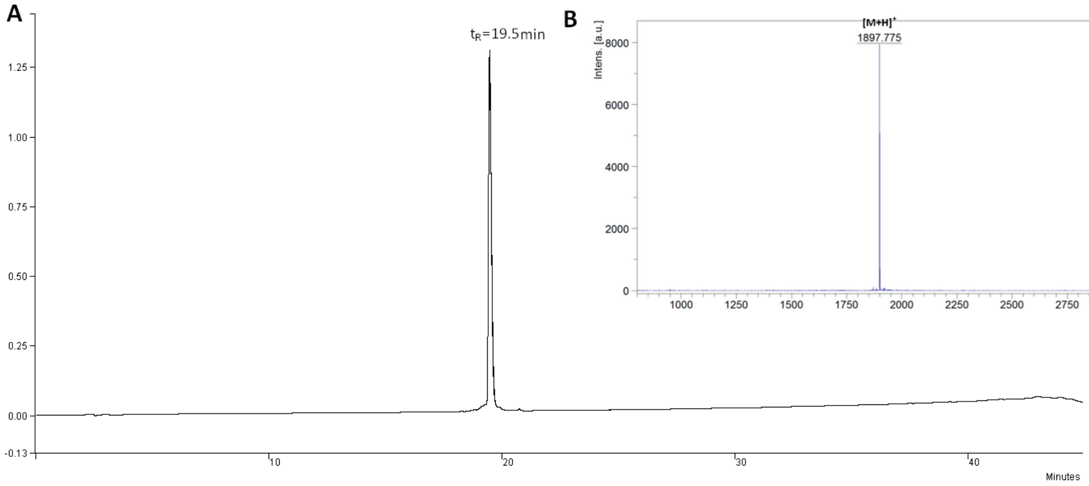

FLCpOH-LFacinB[Nle[1,11]]-NH_2

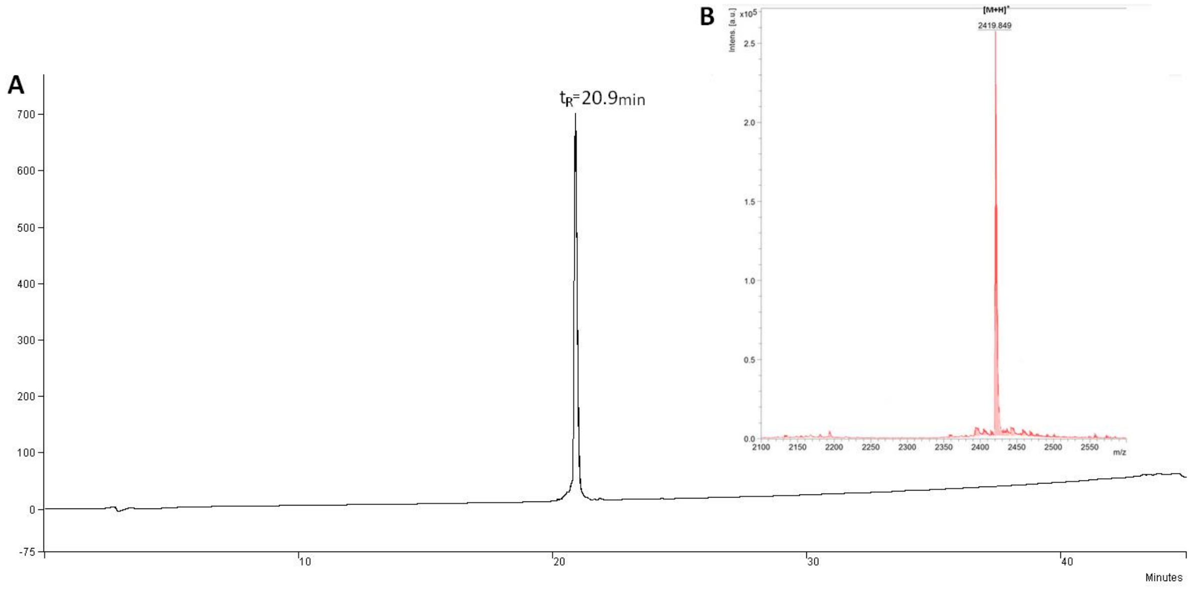

FLCpOH-TP10-7-NH2

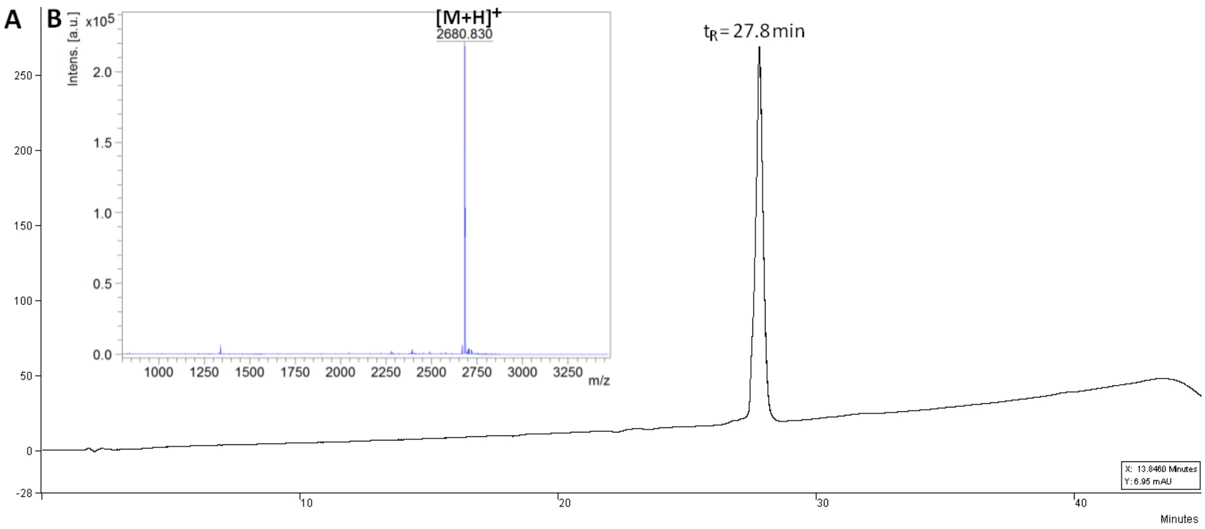

FLCpOH-TP10-NH2

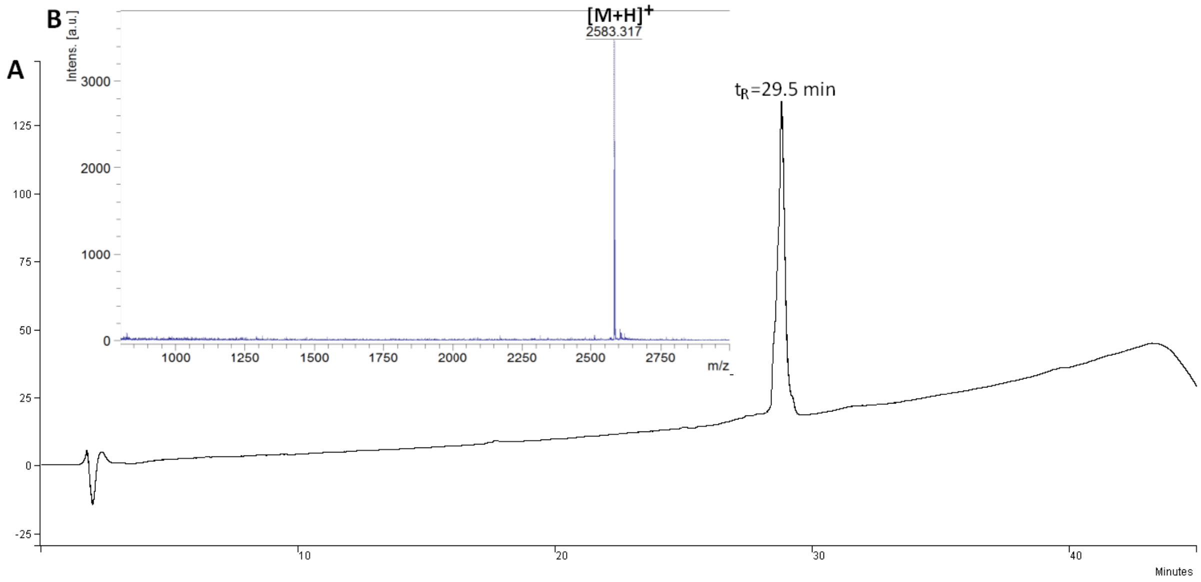

FLCpOH-HLopt2-NH2

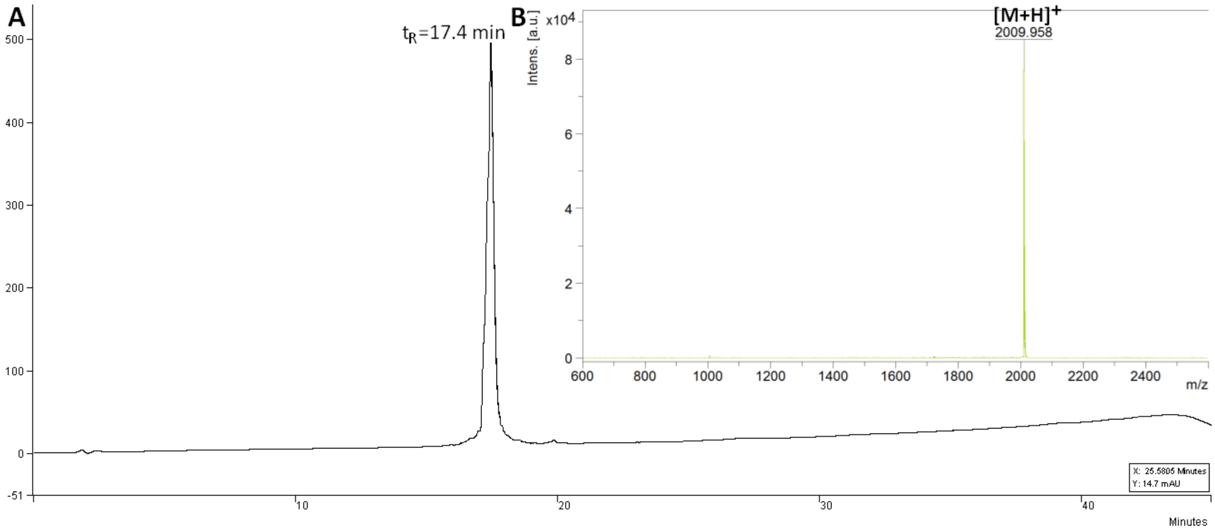

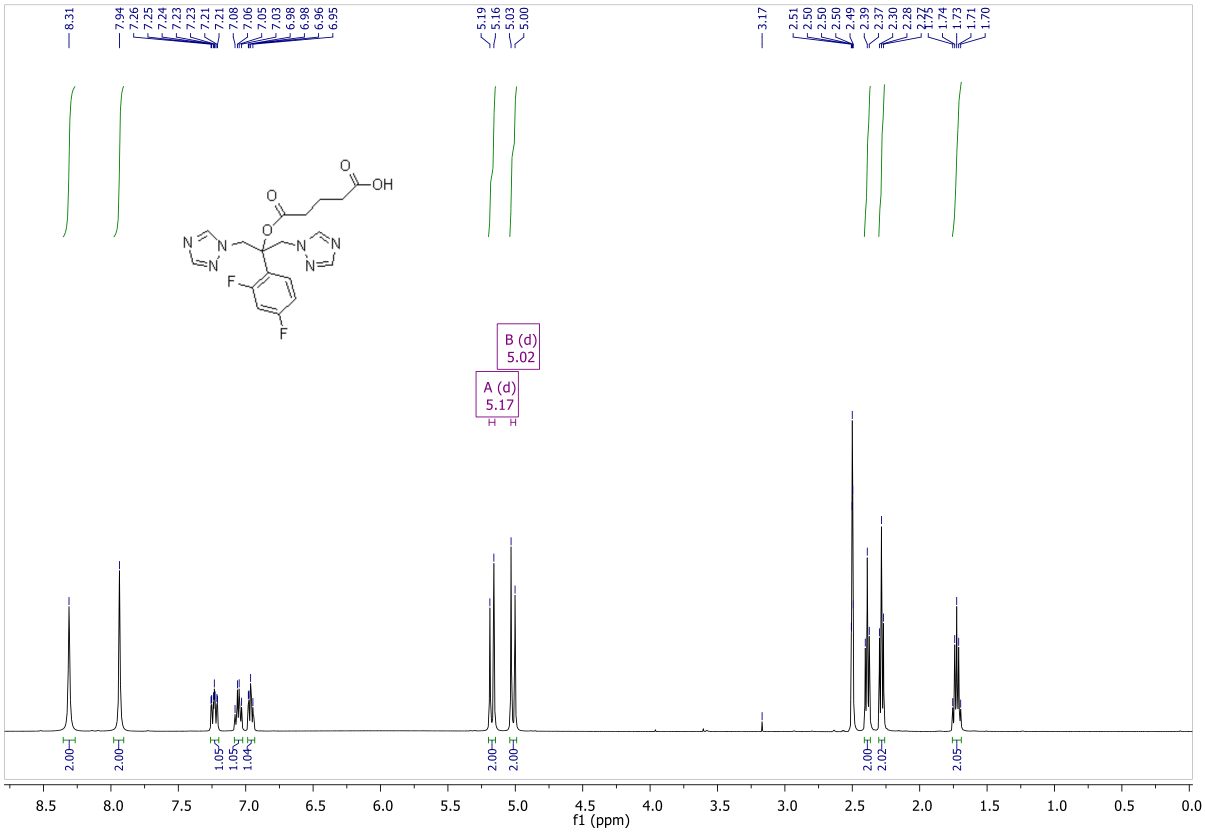

Figure S1. HPLC chromatograms (**A**) and MS analysis (**B**) of the peptide conjugates and their constituents.

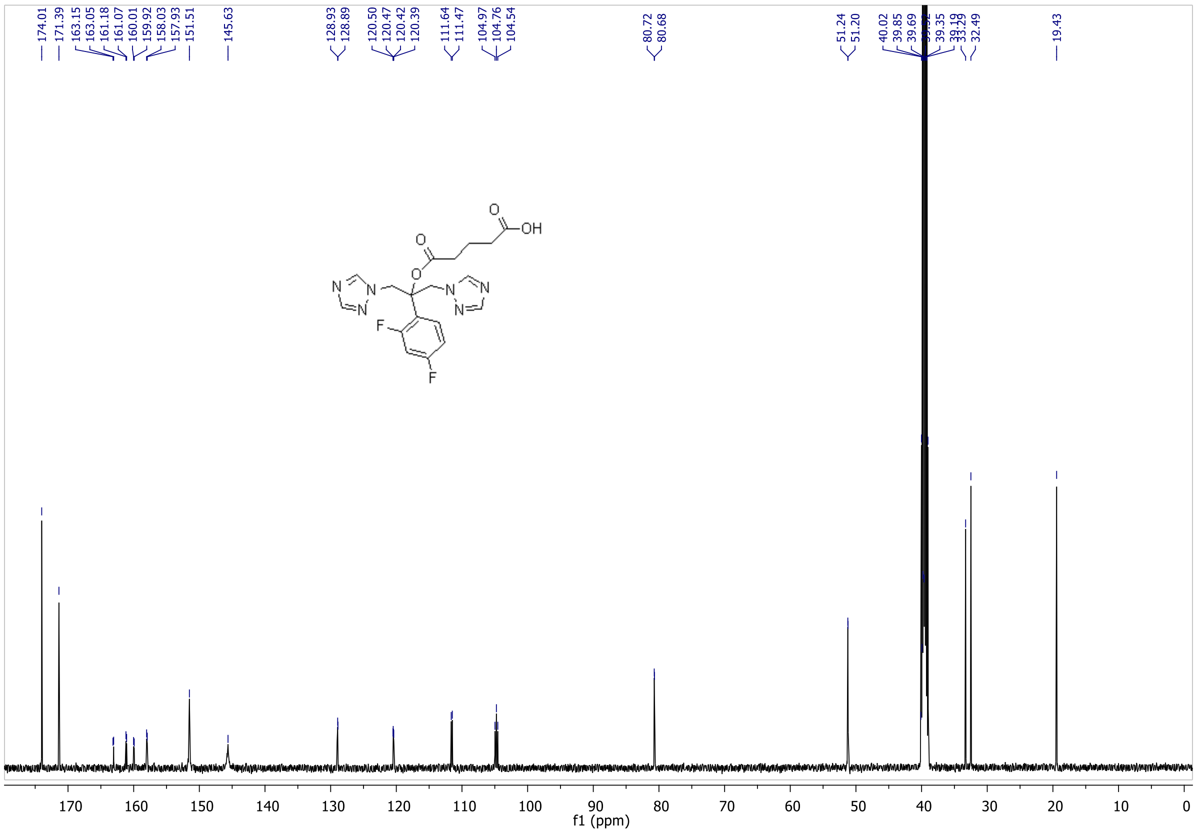

The NMR spectrums were processed in program MestReNova version 6.0.2-5475, Mestrelab Research S,L. Chemical shift (δ) are expressed according to dimethyl sulfoxide (DMSO) in ppm values and coupling constants (J) are expressed in hertz (Hz). The ^{1}H NMR spectrum is showed as: δ chemical shift / ppm (assignation, multiplicity, coupling constant, proton number). ^{13}C NMR spectrum is showed as: δ chemical shift / ppm (assignation). The peaks are marked as: s (singlet), d (dublet) or m (multiplet). The spectrum was analyzed according to one dimensional (^{1}H and ^{13}C). The structure of compound was generated by ChemDraw Ultra (version 12.0.2.1076).

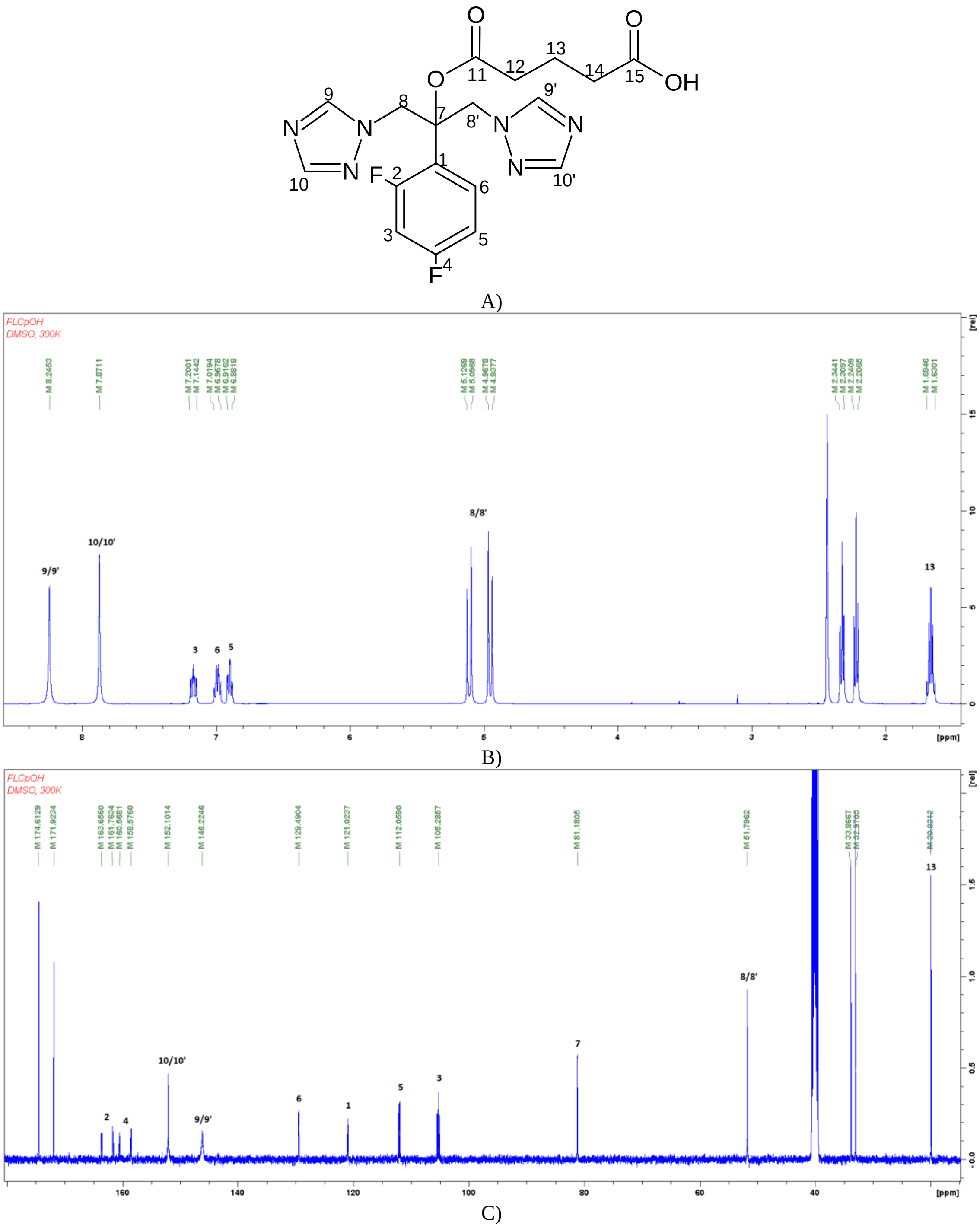

Figure S2. NMR spectrum of the FLCpOH (**A**), ^{1}H NMR (**B**) and ^{13}C NMR (**C**).

Table S1. The antifungal activity of peptides and conjugates in RPMI (200 – 0.4 μg/mL range).

Compound	MW [g/mol]	MIC [μg/mL] <RPMI-1640>													
		C. albicans ATCC 10231		*C. albicans* SC5314		*C. glab*rata DSM 11226		*C. krusei* DSM 6128		*C. albicans* opt1-opt5Δptr2Δptr22Δ		*C. tropicalis* (clinical)		*C. parapsilosis DSM 5784*	
		MIC 90	MIC 50	MIC 90	MIC 50	MIC 90	MIC 50	MIC 90	MIC 50	MIC 90	MIC 50	MIC 90	MIC 50	MIC 90	MIC 50
TP10-NH_2	2180.4	200	200	50	50	200	200	50	50	25	25	ND	ND	ND	ND
HLopt2-NH_2	1606.0	> 250	> 250	ND	ND	> 250	> 250	250	250	ND	ND	> 250	> 250	ND	ND
LFcinB(2-11)-NH_2	1494.8	> 100	100	ND	ND	> 100	> 100	100	50	ND	ND	> 100	> 100	> 100	12.5
LFacinB[$Nle^{1,11}$]-NH2	2016.2	> 100	> 100	ND	ND	> 100	> 100	100	100	ND	ND	> 100	50	> 100	> 100
FLC-COOH	364.1	> 100	> 100	ND	ND	50	25	> 100	> 100	ND	ND	> 100	> 100	> 100	> 100
FLC-CO-TP10-NH_2	2526.5	> 200	>200	50	50	> 200	> 200	> 200	>200	50	25	ND	ND	ND	ND
FLC-HLopt2-NH_2	1953.9	> 250	> 250	ND	ND	> 250	> 250	> 250	> 250	ND	ND	125	125	ND	ND
FLC-LFcinB(2-11)-NH_2	1840.9	> 100	> 100	ND	ND	> 100	> 100	50	25	ND	ND	> 100	> 100	100	12.5
FLC-Nle-LFcinB	2363.3	> 100	100	ND	ND	> 100	> 100	> 100	> 100	ND	ND	> 100	> 100	>100	100
FLC-Nle-LFcinB-NH_2	2362.3	100	50	ND	ND	25	12.5	100	100	ND	ND	> 100	> 100	100	6.25

ND-not determined.

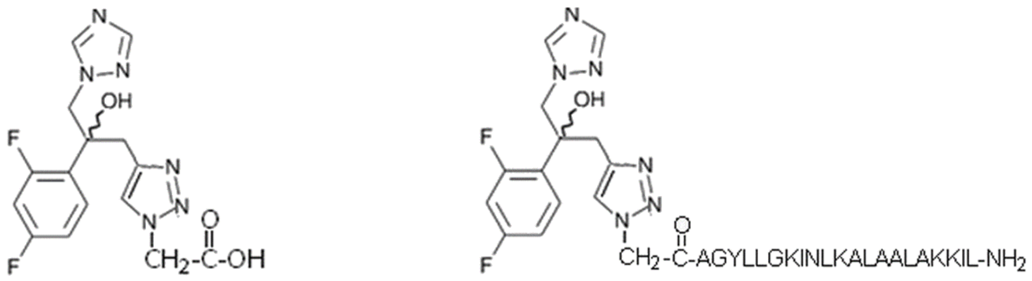

FLC-COOH **FLC-CH_2CO-TP10-NH_2**

Figure S3. The chemical structure of fluconazole derivative (FLC-COOH) and its conjugate with TP10-NH_2.

Table S2. Selectivity indexes (SI) of the FLCpOH-TP10-NH_2 and FLCpOH-TP10-7-NH_2 compounds. The selective activities of the FLC-conjugates were calculated according to the following formula "Selectivity index (SI) = (IC_{90} in μM after 72h) / (MIC_{50} in μM after 24h).

Yearst strains	Hs27		HUVEC	
	**FLCpOH-TP10-NH_2**	**FLCpOH-TP10-7-NH_2**	**FLCpOH-TP10-NH_2**	**FLCpOH-TP10-7-NH_2**
Candida glabrata DSM 6128	0.46	0.15	0.55	0.18
Candida krusei DSM 11226	0.46	0.96	0.55	1.10
Candida albicans SC 5314	3.60	3.72	4.29	4.25
Candida albicans ATCC 10231	1.92	1.98	2.29	2.27

Candida albicans B3	0.92	0.96	1.12	1.10
Candida albicans B4	0.46	0.66	0.55	0.77
Candida albicans Gu4	0.64	1.29	0.76	1.48
Candida albicans Gu5	0.46	0.96	0.55	1.10
Candida albicans 48	0.64	1.99	0.76	2.27
Candida albicans 138	0.64	1.29	0.76	1.48
Candida albicans 190	0.64	1.29	0.76	1.48
Candida albicans 247	0.64	1.29	0.76	1.48
Candida albicans 574	0.64	1.29	0.76	1.48
Candida albicans 604	0.64	1.29	0.76	1.48
